# Enhancing the capabilities of large language models for API calls through knowledge graphs

**DOI:** 10.1038/s41598-026-47696-z

**Published:** 2026-04-10

**Authors:** Ye Yang, Xue Xiao, Ping Yin, Taotao Xie, Yang Ye

**Affiliations:** INSPUR GROUP CO., LTD, No. 1036, Langchao Road, High-tech Zone, Jinan City, 250101 Shandong Province China

**Keywords:** Large Language Models, API calls, Knowledge Graphs, Meteorology, Engineering, Mathematics and computing

## Abstract

API calls by large language model (LLM) represent a cutting-edge technique in data analysis. However, the potential of LLM to effectively utilize tools through API calls remains underexplored in knowledge-intensive sectors such as the meteorological industry. In this paper, we propose a system, named KG2data, that integrates knowledge graphs, LLM, React agents, and tool usage technologies to perform API calls for intelligent data acquisition and query handling in the meteorological domain. We test the accuracy of the system’s API calls using a virtual API. The baseline systems for comparison are chat2data (KG2data without knowledge) and RAG2data (KG2data with a vector database replacing the knowledge graph). Our experimental results demonstrate that the proposed system (1.43%, 0% and 88.57% in 3 evaluation metrics) outperforms RAG2data (16%, 10% and 72.14% in 3 evaluation metrics) and chat2data (7.14%, 8.57% and 71.43% in 3 evaluation metrics) in terms of failure rate of name recognition, failure rate of hallucination recognition and accuracy rate for API calls. Our system integrates knowledge graph, LLM, and ReAct-master agent technologies. Unlike current LLM used for API calls, our system overcomes the challenge of limited domain-specific knowledge of LLM, which often makes it difficult to address complex queries containing specialized terminology or lengthy questions. By utilizing knowledge graphs as long-term memory, our system significantly improves conten-retrieval coverage, handling of complex queries, industry-specific logical reasoning, deep semantic relationships among entities, and the integration of heterogeneous data. Additionally, it addresses the high computational costs associated with training or fine-tuning LLM, making it more adaptable to the dynamic nature of domain knowledge and APIs. In summary, the KG2data system offers a fresh perspective for intelligent knowledge-based question answering and data analysis in knowledge-intensive industries.

## Introduction

### Impacts of extreme weather and demands for accurate meteorological services

With the increasing frequency of climate change and extreme weather events (droughts, floods, heatwaves, storms), various industries are facing far-reaching and persistent impacts (Bank for International Settlements, 2025). Specifically, agriculture, forestry and fishing suffer severe production losses from droughts and floods that threaten food security; the energy and water sectors face supply volatility due to heatwaves and water shortages; manufacturing and construction experience supply chain disruptions and reduced labor productivity; and transportation, healthcare, and financial services are indirectly affected through infrastructure damage, elevated public health risks, and surging insurance claims^1–3^. In order to effectively address these challenges, accurate meteorological services have become crucial, which poses challenges to the processing and application of large-scale meteorological data. With the rapid advancement of new-generation information technologies such as large language models (LLMs), cloud computing and big data, new possibilities have been created for precision meteorological services. Nevertheless, the high barriers to application, massive demand for computing resources and substantial investment costs of these technologies have brought severe challenges to the original meteorological data systems^4–6^.

### Limitations of traditional meteorological data utilization

The traditional form of meteorological data utilization has significant limitations, mainly manifested in the following aspects:

Difficulty in effective utilization of data: Meteorological data is highly diverse and complex, and traditional data modeling methods are difficult to process and analyze these data efficiently; it is difficult to sort out the data relationships and it depends on human experience, further increasing the operational difficulty^7^.

Data confidentiality issue: Meteorological data involves national security, military use and economic interests, and sensitive data restricts public sharing and commercial application^8^.

Data valuation problem: The large-scale demand, high-value application and technological accumulation of meteorological data in China provide good market and supply conditions for meteorological data services to release their high value. However, the value of meteorological data elements has not formed a standardized and normalized system of value evaluation^9,10^.

Challenges in addressing the diverse needs of multi-industry applications: commercial meteorological services have reached a mature stage in Europe and the United States, generating annual revenues in the hundreds of billions of U.S. dollars. In contrast, this industry in China remains in its nascent phase, yet it holds significant growth potential. The vast scale and diversity of meteorological data present considerable challenges in data retrieval, particularly for non-expert users.

### Limitations of traditional machine learning and advantages of LLM in data system optimization

Over the past decade, machine learning, such as Learning-driven optimizer^11^, learned index^12^, and Intelligent tuning of database parameter configuration^13^, have been widely applied across various industries to enhance data systems, aiming to reduce user barriers and improve efficiency. However, these methods exhibit a high dependency on training data, limited generalization capabilities, and challenges in adapting to real-time adjustments in data systems as they evolve over time. Since 2022, the gradual maturation of generative large language model (LLM), such as GPT, has demonstrated impressive performance in understanding user intent and generating task-specific outputs. In particular, these models have shown significant potential in optimizing data systems and improving data governance^14–16^.

### Challenges of LLM in domain-specific data query and API call applications

Despite the superior performance of LLM in many fields, challenges remain in leveraging these models for automated, intelligent, and precise data querying, recommendation, and user-specific analysis. Key challenges include hallucinations^17,18^ due to the lack of domain-specific knowledge, high inference costs associated with LLM, and inaccurate reasoning results for tasks requiring high precision^17^.

To address these challenges, some studies have enabled LLMs to utilize external tools^19^, allowing them to access larger and dynamically evolving knowledge bases and perform a wide range of subtle tasks. Building upon this, the interaction between LLM and external APIs has gradually become a research hotspot. By providing access to computational tools, research by^20^ has demonstrated that enhanced LLM can handle larger, more dynamic knowledge spaces and perform complex computational tasks. Consequently, leading LLM providers^21^ have begun integrating plugins to enable these models to call external tools via application programming interfaces (APIs). This integration allows users to invoke complex software functions through simple inputs, thereby improving interaction efficiency and lowering the barriers to software usage.

### Limitations of Current LLM-API integration in specialized domains

However, many previous studies integrating APIs into large language models^22^ have seldom considered the system’s performance in specialized domains, such as meteorology. In fact, in the context of domain-specific tasks, user queries are often semantically implicit and contain substantial background information. Such semantically ambiguous queries may lead large language models (LLM) to hallucinate responses. Moreover, due to the lack of domain expertise, LLM may struggle to fully understand user intentions within specialized fields. Lastly, domain-specific knowledge is inherently dynamic, and LLM, due to the high computational cost of retraining and fine-tuning, typically find it difficult to adapt to the evolving nature of both domain knowledge and APIs. These three challenges make the standard approach of using LLM for API calls difficult to adapt to specialized domains.

### Limitations of RAG2data and advantages of knowledge graphs in domain-specific tasks

In this context^23^, have innovatively proposed a conversational, interactive data analysis platform driven by Large Language Models (LLM) and Retrieval-Augmented Generation (RAG) techniques, referred to as RAG2Data. RAG2Data leverages RAG technology to manage unstructured data within specialized domains, while structured data is retrieved via a Text-to-SQL approach. This design significantly reduces the need for direct interaction with LLM, mitigating issues such as hallucinations, low reasoning accuracy, and high inference costs commonly associated with LLM. However, the vector similarity search mechanism of RAG presents several challenges in current applications: (1) the retrieval coverage is limited, making it difficult to uncover deep semantic relationships between data; (2) it struggles to perform complex queries; (3) it cannot effectively integrate heterogeneous data from diverse sources; (4) it fails to perform reasoning and extension of knowledge in specialized domains; and (5) it suffers from delays in data updates.

Recent studies have focused on combining Large Language Models (LLM) with knowledge graphs to build interactive question-answering knowledge bases tailored to specialized domains, aiming to address the limitations of Retrieval-Augmented Generation (RAG) technology^24–26^. A knowledge graph serves as a structured framework for representing knowledge relationships. Its structured associative architecture allows for the integration of datasets from diverse sources^27,28^. This facilitates deep semantic relationship mining^29^ and knowledge reasoning extension within specialized domains^30,31^. Moreover, knowledge graphs support complex queries, such as path and subgraph queries, and have shown strong performance in knowledge-intensive tasks^32^. These advantages are not inherent to RAG technology. Consequently, leveraging knowledge graphs to enhance LLM performance in specialized tasks has become a significant trend in current development.

### Research motivation and proposed KG2Data system

Currently, few technologies leverage knowledge graphs to enhance the performance of large language models (LLM) in specialized tasks, such as the precise retrieval, analysis, and processing of data within the context of expert knowledge semantics. To address the following challenges: (1) the inefficiency in meteorological data acquisition and utilization, as well as data confidentiality issues, (2) the limitations of Retrieval-Augmented Generation (RAG) technology in performing complex queries and its lack of knowledge reasoning capabilities, and (3) the absence of domain-specific expertise in current LLM-based API calling methods, our team proposes an innovative system, **LLM-Driven Meteorological KG2data**. This system integrates knowledge graph technology, React-based expert agent technology, LLM, and API calls to achieve intelligent, automated meteorological data acquisition and analysis within a legal and compliant framework.

## Related work

### LLM for tool usage

Large Language Models (LLM) have made significant advancements in the field of natural language processing. The concept of LLM for Tool Usage refers to leveraging LLM to interact and collaborate with various tools to accomplish more complex tasks and solve real-world problems. These tools can include, but are not limited to, the following types:

External Databases and Knowledge Graphs: LLM can access external databases and knowledge graphs to obtain more accurate and comprehensive information, thereby enhancing the quality of responses and text generation. For example, when addressing domain-specific queries, LLM can utilize specialized databases to retrieve the latest research findings and data^33^.

Software Tools and APIs: Integration with various software tools and application programming interfaces (APIs) enables LLM to perform specific and subtle tasks, such as data analysis and image recognition. For instance, by invoking mathematical function libraries, LLM can carry out complex mathematical computations^34^.

Agents and Automation Systems: As agents, LLM can collaborate with other automation systems to execute tasks autonomously and optimize workflows. For example, in software development, LLM can interact with agents and tools to enhance development efficiency^35^.

### Conversational search and contrastive retrieval

Conversational search has established itself as a core paradigm for next-generation information access, characterized by its ability to handle multi-turn interactions, maintain contextual awareness, and process complex user intents through natural language dialogue^36^. In a comprehensive survey^36^, systematically reviewed the key components of conversational search systems, including query reformulation, conversational retrieval, and response generation, emphasizing the transformative role of LLMs in enabling robust information integration and context management for intricate queries.

Contrastive retrieval, as an optimized retrieval methodology, has demonstrated superior performance in distinguishing fine-grained semantic differences in unbalanced data scenarios, which provides valuable technical insights for improving the accuracy of domain knowledge retrieval in LLM-based API call systems^37^. advanced this field by proposing a retrieval-augmented contrastive learning framework tailored for fine-grained semantic discrimination in low-resource and unbalanced settings. By adapting contrastive learning to distinguish between literal and figurative meanings, their work validated that dense retrieval combined with contrastive objectives can achieve high recall and strong generalization in real-world scenarios^37^. This methodology is particularly instructive for our domain-specific task, where precise discrimination between semantically similar meteorological concepts is critical to avoid API invocation errors.

The design philosophy of conversational search—supporting implicit and complex user queries with contextual understanding and robust information processing—aligns with the core demand of domain-specific API call tasks (e.g., meteorological API calls) for accurate interpretation of professional, implicitly framed user inquiries, and provides a foundational theoretical reference for building interactive LLM-driven API call systems that can handle multi-turn and complex domain queries.

### LLM for API calls

API calls have become a cutting-edge focus in the application of LLM, with increasing encouragement to use them as tools^38,39^. This technology enables users to retrieve and process data through natural language queries, eliminating the need for complex programming knowledge. By transforming natural language into a JSON format that meets the specific requirements of APIs, the data analysis process becomes more intuitive and efficient^40^. However, limited research has been conducted on API calls in vertical domains, which are crucial for the precise acquisition and analysis of specialized data.

### RAG2data

RAG2data, proposed by^23^, is a data analysis platform enhanced by Large Language Models (LLM) and Retrieval-Augmented Generation (RAG) techniques. The architecture of RAG2data comprises three layers. The knowledge management layer is responsible for collecting and preprocessing data, splitting it, selecting embedding models, storing it in a vector database, and managing tools. The online query inference layer handles query preprocessing, converts queries into vectors, analyzes intent, and either retrieves knowledge and APIs for single-round LLM input or utilizes an LLM agent for multi-round pipeline generation. This layer also leverages the vector database for caching purposes. The LLM layer generates results, summarizing unstructured data, translating natural language into SQL, and employing pandas APIs for structured data analysis and visualization.

Previous research has largely overlooked the performance of API calls in specialized domains, either due to challenges in data acquisition within these fields or a lack of knowledge inference capabilities specific to the domain. Additionally, the high cost of training and fine-tuning large language models (LLM) makes it difficult for them to adapt to the dynamic changes in APIs and domain-specific knowledge. In contrast, knowledge graphs offer a promising solution to address these limitations.

Unlike previous studies, our research focuses on a more constrained domain—API calls in specialized fields, exemplified by atmospheric science. By integrating knowledge graphs, agents, LLM, and tool usage, we enable API calls that enhance the performance of LLM in domain-specific data acquisition and analysis, without the need for complex fine-tuning or dealing with low-level implementation details.

## Methodology

In this section, we describe the construction process of KG2data and the associated technologies. First, we outline the process of preparing the API dataset and the creation of instruction-answer pairs used for technical performance evaluation. Subsequently, we introduce KG2data, an innovative framework driven by Large Language Models (LLM) that integrates domain-specific knowledge graphs to facilitate domain-specific API calls. Finally, we present the matching evaluation metric employed to assess the system’s performance in executing API calls.

### Dataset collection

#### API documentation

This study constructs a Function Calling capability testing framework for the meteorological domain. To systematically evaluate the tool invocation capability of LLMs in professional meteorological scenarios, a comprehensive testing system covering basic meteorological elements to professional analysis scenarios is established by combining simulated meteorological API interfaces with real business instruction-answer pairs.

In terms of API design and implementation, 35 meteorology-related API interfaces are designed and encapsulated as agent tool functions. These functions adopt a standardized interface design, uniformly receiving character strings in JSON format as input parameters and returning structured meteorological data after parsing. All functions are implemented using Mock data, i.e., the return results of real meteorological APIs are simulated through predefined JSON data, rather than invoking external meteorological service interfaces in practice. This design choice is mainly based on the following considerations^41^: First, real meteorological APIs are usually subject to invocation limits, cost expenses and data timeliness issues, which are not conducive to large-scale repeatable experiments. Second, Mock data can ensure the reproducibility of test results and avoid fluctuations in experimental outcomes caused by changes in external data sources. Third, predefined data allows for precise control of test scenarios and the construction of complete test cases including normal, boundary and abnormal conditions.

Specifically, the implementation of meteorological APIs covers meteorological elements at multiple levels. Basic meteorological elements include get_2m_temperature (2-meter height temperature), get_mean_sea_level_pressure (mean sea level pressure), get_total_precipitation (total precipitation), get_10m_wind_speed (10-meter height wind speed), get_relative_humidity (relative humidity) and other functions, which simulate the acquisition of conventional surface meteorological observation data. Intermediate meteorological elements cover professional observation items such as get_soil_temperature (soil temperature), get_soil_moisture (soil moisture) and get_surface_radiation (surface radiation). Advanced meteorological analysis elements include key parameters for severe weather analysis such as get_convective_available_potential_energy (Convective Available Potential Energy) and get_lightning_observations (real-time lightning observations). In addition, interfaces for public services are implemented, such as get_climate_and_temperature (query of climate type and annual average temperature), get_city_weather_forecast (urban weather forecast), get_weather_and_alert (weather conditions and meteorological early warnings), get_typhoon_info (typhoon information) and so on, so as to cover a complete application spectrum from professional research to public services.

**Fig. 1:**
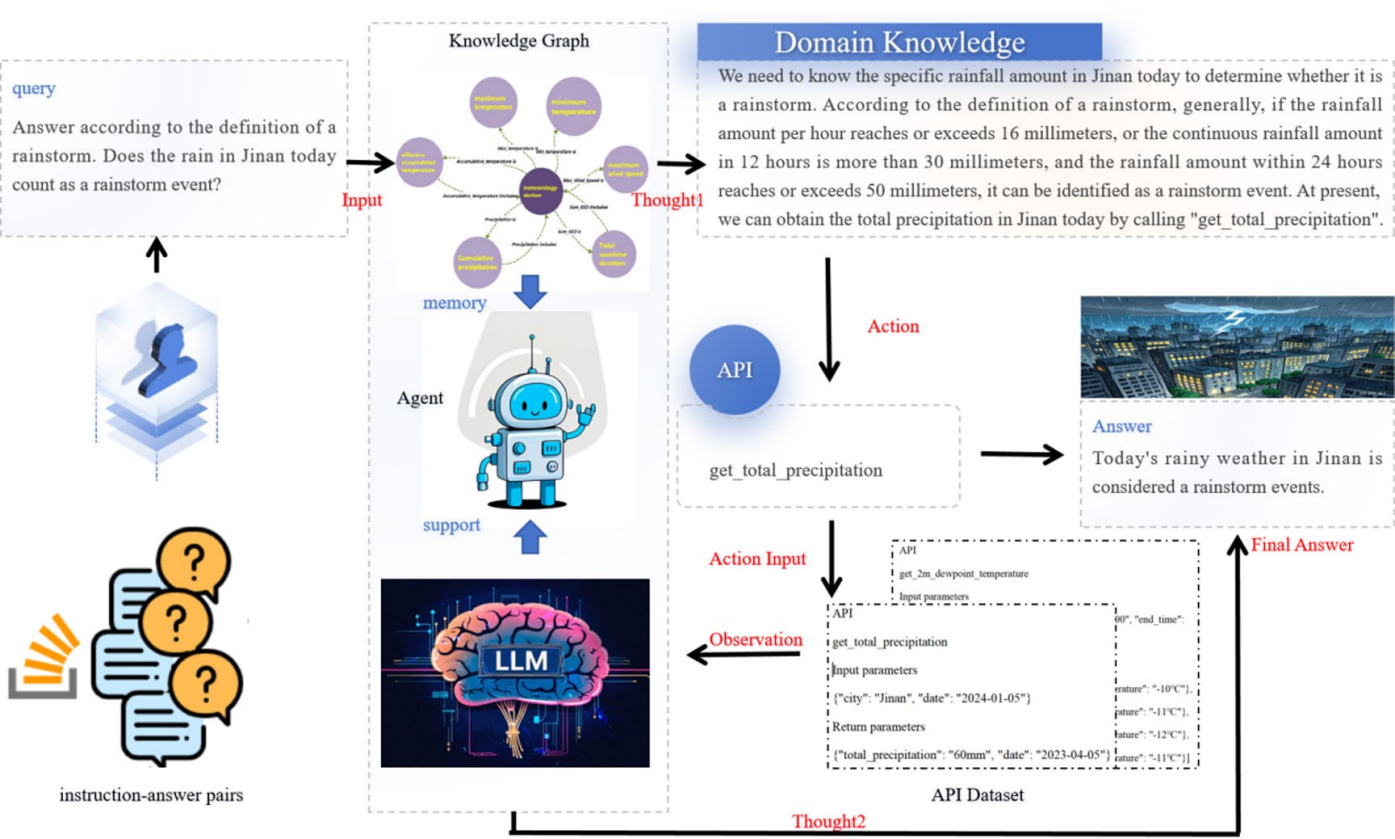
KG2data: an interactive data analysis system that integrates a knowledge graph, an agent, and a large language model (LLM) to facilitate interactions with API. The lower half of the system illustrates the preliminary preparation of API datasets and instruction-answer pairs, as outlined in Sect. 3.1 .The upper half depicts the architecture and operational process of KG2data, as detailed in Sect. 3.2. In this example, the system is capable of suggesting the appropriate API calsl to generate answers from a user’s natural language query.

#### Instruction-answer pairs

The instruction-answer pair dataset constructed in this study has a one-to-one correspondence with the self-designed APIs: each instruction-answer pair is explicitly matched to a specific meteorological tool function and its parameter specifications^42^. The user query expressions cover the typical invocation scenarios of each tool, and the expected answers as well as intermediate derivation steps are based on the return structure of the corresponding API. Thus, this dataset can be used to directly verify whether the model can correctly select and invoke the meteorological APIs designed in this study.

To balance the research depth in professional scenarios and the coverage breadth in general scenarios, the design of questions in this study strictly follows the demand characteristics of real meteorological business and incorporates a large number of professional terms and standardized expressions, involving core meteorological concepts such as the subtropical high at the 500 hPa level, the wind shear line at the 850 hPa level, Convective Available Potential Energy (CAPE), and water vapor flux divergence. The questions cover a gradient of complexity, ranging from simple single-element queries (e.g., “What are the climate type and annual average temperature of Jinan?“) to complex multi-element comprehensive analysis questions (e.g., “Analyze the relationships among the 500 hPa height field, 850 hPa wind field, temperature and shear line during the rainstorm process in Pingyin on May 17, 2008”). All professional questions are constructed with reference to meteorological operation manuals, which ensures their professionalism and practical application value.The construction of professional questions is critical for reducing hallucinations and enhancing the accuracy of tool invocation^42^.This is of paramount importance, as highly specialized phrasing in questions posed by domain experts may be too complex for LLM to comprehend, potentially impacting the API calls.

#### Data sources and reference standards

Although Mock data is adopted in the actual implementation, the design of APIs and their data structures are all based on authoritative international and domestic meteorological data standards. For historical meteorological reanalysis data, we refer to the interface specifications of the ERA5 reanalysis dataset from the European Centre for Medium-Range Weather Forecasts (ECMWF). The ERA5 dataset provides hourly reanalysis data of global atmospheric, terrestrial and marine climate variables from 1940 to the present, and its API interface design includes standardized parameters such as parameter codes (e.g., msl for mean sea level pressure, tp for total precipitation), time ranges, altitude levels and spatial resolution. Our functions such as get_mean_sea_level_pressure and get_total_precipitation follow the specifications of ERA5 in terms of parameter naming and data structure design. For real-time and forecast data, we refer to the interface design of the open-source weather forecast API from Open-Meteo (https://open-meteo.com/). Open-Meteo provides free API services based on multiple numerical weather prediction models with a concise interface design and comprehensive documentation, and we simulate the JSON structure of its returned data in our code implementation.

#### Generalization capability for real meteorological apis and actual user queries

Generalization to Real Meteorological APIs.

The current design is primarily based on Mock meteorological APIs and tools. Since the tool interfaces follow the standards of ERA5, Open-Meteo and other authoritative platforms in parameter naming and return structures, the models’ abstract capabilities in tool invocation and returned data parsing can be transferred to real APIs with similar structures. In other words, given the close similarity between the request/response formats of real meteorological APIs and the existing Mock design, the models can maintain largely consistent performance in tool selection, parameter configuration and result interpretation when facing real APIs.

Generalization to Actual User Queries.

At the user query level, the current data construction method also lays a solid foundation for generalization. The professional questions constructed based on meteorological operation manuals cover common professional terms and complex query expressions in practical meteorological work, which is conducive to the improvement of the models’ intent recognition and tool selection capabilities in professional meteorological scenarios. Therefore, the models demonstrate certain generalization potential in function selection and parameter configuration for queries that are consistent with the training/test data distribution, especially for standardized or semi-standardized queries in the meteorological domain.

### KG2data

#### System overview

This study proposes a KG2data framework driven by LLMs to enhance the capability of API calling in the atmospheric science domain. The system consists of four core modules: a meteorological knowledge graph, a ReAct expert agent, a large language model (LLM), and a callable set of meteorological data tools. The overall system architecture is shown in Fig. 1. Unlike conventional API calling methods that rely solely on function descriptions, this framework retrieves domain knowledge from a structured knowledge graph to support the agent’s reasoning, enabling it to select appropriate APIs in a professional meteorological context and generate reliable answers integrated with expert knowledge. Detailed implementation descriptions of each module are provided below to ensure the reproducibility of the system.

#### Knowledge graph

Data source and preprocessing.

The corpus for the knowledge graph is derived from meteorological operational reference manuals (the names and detailed information of the manuals are not disclosed per the requesting institution’s requirements). These manuals cover severe weather phenomena such as rainstorms, typhoons, hailstorms and heavy fogs, as well as their formation mechanisms, forecasting indicators and historical cases. In this study, Qwen-Turbo is adopted as the backbone LLM to automatically segment the corpus into semantically coherent text chunks, with each chunk containing approximately 50–300 Chinese characters.

Entity and Relation Extraction.

The in-context learning capability of LLMs is utilized to extract entities and relations from each text chunk. Specifically, a system prompt is designed to instruct the model to act as a meteorological professional and output knowledge triples in the form of a 5-tuple (head entity, head entity type, relation, tail entity, tail entity type). The system prompt template for entity-relation extraction is as follows:

System Prompt: You are a meteorological professional working at the National Meteorological Center, tasked with building a knowledge graph database.Your job is to extract information from the given text and convert it into knowledge graph data.Please extract meaningful relations from the text as comprehensively as possible, including:

Explicit direct relations stated in the text;

Reasonably inferable implicit relations;

Attribute relations describing entity properties;

Temporal and causal relations between events;

Whole-part relations between systems and their components.

Please provide the node set in the format of [head, head_type, relation, tail, tail_type].The output must be a list in JSON format, where each object contains the keys: “head”, “head_type”, “relation”, “tail”, “tail_type”.

To guide the LLM to perform accurate extraction in the meteorological domain, 13 manually annotated few-shot examples are provided, covering various entity types (e.g., atmospheric systems, weather phenomena, physical quantities, equipment, formulas) and relation types (e.g., “influences”, “causes”, “occurs in”, “observes”). The entity and Relation types schema are shown in Tables 2 and 3, respectively.

After the initial extraction, the Leiden algorithm is applied to perform multi-level community detection on the knowledge graph. This step serves two purposes: (1) identifying semantically coherent entity clusters (e.g., all entities related to typhoon forecasting); (2) pruning redundant or low-confidence edges to reduce retrieval noise. The pruned knowledge graph is stored in a Neo4j graph database, and a vector index is established to support similarity retrieval.

Knowledge Graph Storage.

The final configuration of the knowledge graph in Neo4j is as follows:

Embedding model: BGE-M3 (1024-dimensional vector), deployed locally via Ollama.

Retrieval query: multi-hop traversal starting from matched entities, with a maximum hop count of 3.

#### ReAct expert agent

LLM Configuration.

The ReAct expert agent adopts Qwen-Turbo (Tongyi Qianwen) as the reasoning engine. Developed by Alibaba Cloud, this model is invoked via the DashScope API. The model specifications are as follows:

Model name: qwen-turbo.

Service provider: Alibaba Cloud DashScope.

Context window: 8,000 tokens.

A hybrid scheme is adopted for text embedding:

BGE-M3: deployed locally via Ollama for knowledge graph entity embedding;

text-embedding-v2: invoked via the DashScope API for document chunk embedding.

ReAct Framework Implementation.

The agent follows the ReAct (Reasoning and Acting) paradigm, interleaving reasoning trajectories with tool calls. The structure of the reasoning chain is: [Input] → [Thought1] → [Action] → [Action Input] → [Observation] → [Thought2] → [Final Answer].

The hyperparameter settings for the ReAct agent are:

Maximum number of iterations: 10.

ReAct Agent System Prompt.

The system prompt received by the agent defines its role, available tools and output format requirements. The core content is as follows:

System Prompt (Excerpt): You are a professional meteorological operational assistant. Your input includes the user’s question and professional knowledge retrieved from the knowledge base.You must execute the task in strict accordance with the following steps:

Thought1: Analyze and determine which tools (multiple tools allowed) are needed to acquire data for answering the question.

Action: Select the tool name(s).

Action Input: Input parameters in JSON formatObservation: The results returned by the tools (must be copied verbatim, no fabrication allowed).

Thought2: Summarize the results in human-readable language, which must include the numerical results from the Observation and the professional knowledge from the input.

Final Answer: Present the result to the user.

Available Tools:

get_climate_and_temperature — Query climate type and annual average temperatureInput: {“city”: “City Name”}Parameter examples: {“city”: “Beijing”}, {“city”: “Jinan”}, {“city”: “Kunming”}, {“city”: “Hangzhou”}, {“city”: “Yantai”}

get_typhoon_info — Query typhoon information and path predictionInput: {“region”: “Region Name”}Parameter examples: {“region”: “Shanghai”}, {“region”: “Guangzhou”}, {“region”: “Fujian”}, {“region”: “South China Sea”}, {“region”: “East China Sea”}…[A total of 35 tools are listed with their descriptions and parameter schemas].

Knowledge-Enhanced Reasoning.

Before invoking any tool, the agent first queries the knowledge graph to acquire relevant professional knowledge. Retrieval is implemented through Multi-hop cascaded query, with a maximum of 3 hops traversed from matched entities.

The retrieved context (Top-3 entities, relevant document chunks and recommended tools) is concatenated before the user’s question, which the agent uses to determine the APIs to be called.

#### Data acquisition tools

Tool Registry.

The system maintains a registry of 35 meteorological data tools, where each tool is defined with a unified schema containing four attributes:

Name: A descriptive identifier for easy recognition by the LLM (e.g., get_2m_temperature).

Description: A natural language explanation of the tool’s function.

Parameters: Mandatory and optional inputs defined in JSON Schema.

Function binding: The Python function implementing the API call.

Tool Schema Definition.

**Fig. 2:**
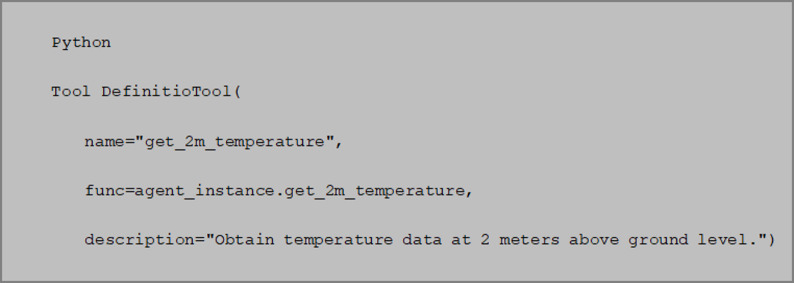
The example of registered tools.

Tools are registered via the Tool class of LangChain, with a structural example as follows (Fig. 2):

Tool implementation example.

**Fig. 3:**
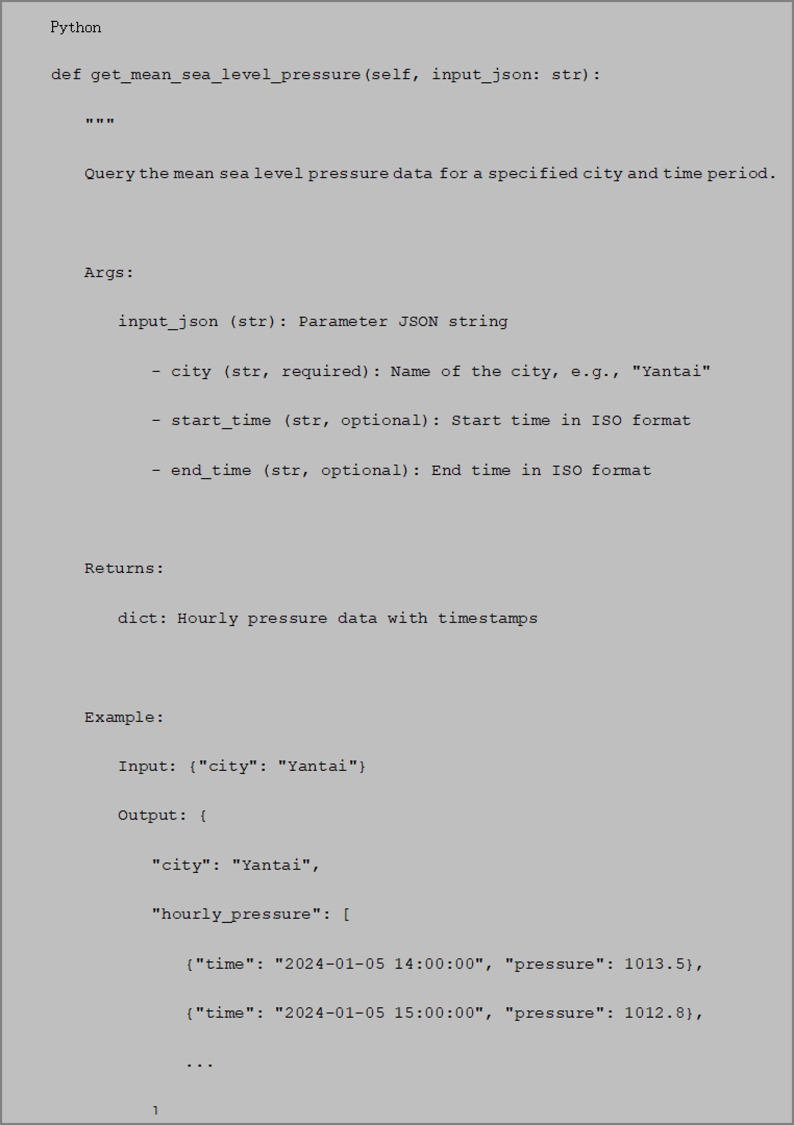
An example of complete tool implementation.

A complete tool implementation example, including parameter schema and simulated return data, is provided in Fig. 3.

Tool Selection Mechanism.

The LLM selects tools based on three criteria: (1) semantic matching between the user’s question and tool descriptions; (2) knowledge graph recommendation: the user’s question triggers knowledge graph retrieval, and highly relevant tools are recommended based on the retrieved edges and entities; (3) parameter satisfiability: whether mandatory parameters can be extracted from the question. The executor verifies the tool selection by checking the consistency between the output action and the parameter names of registered tools; illegal tool names (i.e., hallucinated APIs) are recorded and used for performance evaluation.

For the naming convention adopted in this work, we emphasize the principles of explicitness, consistency, and syntactic simplicity to facilitate rapid intent recognition by LLM agents. Specifically, tool names follow a verb-noun structure that directly maps to the core meteorological task performed (e.g., retrieve_nwp_data, compute_grid_statistics). This design choice eliminates ambiguous modifiers or non-standard abbreviations that often lead to misclassification. By aligning with standard domain terminology, the LLM can efficiently parse the tool semantics, thereby ensuring reliable execution of workflow logic.

### API verification

To systematically evaluate the performance of LLMs on API calling tasks in the meteorological domain, this study adopts a multi-dimensional quantitative evaluation framework. Based on the evaluation system proposed in Reference^39^, we define five core metrics: API Calling Accuracy Rate (ACAR), Failure Rate of Intent Recognition (FRIR), Failure Rate of Name Recognition (FRNR), Failure Rate of Parameter Configuration (FRPR), and Failure Rate of API Hallucination (FRHR). To ensure the precise and mutually independent definition of each metric, we first delineate the judgment scope and calculation logic for each metric, and then present the specific judgment criteria and illustrative examples.

#### Metric definitions

Notation:

$\:N$: Total number of question-answer samples in the test set.

$\:{f}_{i}^{\ast\:}$: Target API name for the i-th sample.

$\:{p}_{i}^{\ast\:}$: Target parameter set for the i-th sample.

$\:{f}_{i}$: Actual API name output by the model.

$\:{p}_{i}$: Actual parameter set output by the model.

$\mathcal{F}$: Set of legally registered APIs in the system.

$\:{N}_{IF}$: Number of samples with failed intent recognition.

$\:{N}_{H}$: Number of samples with hallucinatory outputs.

$\:{N}_{NC}$: Number of samples with correctly selected API names.

$\:\mathbb{I}[\bullet\:]$: Indicator function (returns 1 if the condition is satisfied, 0 otherwise).

Let the test set contain N question-answer samples, where the expected API call for the i-th sample is$\:\langle{f}_{i}^{\ast\:},{p}_{i}^{\ast\:}\rangle$, and the actual API call output by the model is $\:\langle{f}_{i},{p}_{i}\rangle$. The definitions of each metric are as follows:

**API Calling Accuracy Rate (ACAR)**: This metric measures the model’s ability to complete API calls completely and correctly. A sample is marked as a successful call only if the model meets all the following conditions: (1) the model identifies the need to call an API; (2)$\:{f}_{i}={f}_{i}^{\ast\:}$; (3) $\:{p}_{i}\subseteq\:{p}_{i}^{\ast\:}$ with all mandatory parameters included; (4) the API execution returns valid results. The calculation formula is: $\:ACAR=\frac{1}{N}\sum\:_{i=1}^{N}\mathbb{I}\left[Correc{t}_{i}\right]$

**Failure Rate of Intent Recognition (FRIR)**: This metric measures the proportion of cases where the model fails to correctly understand the user’s query intent in the [Thought1] stage. Specifically, intent recognition is deemed failed (denoted as $\:{IF}_{i}$) if the model’s reasoning process fails to identify the need for an external API call, or if there is a fundamental deviation between the model’s understanding of the question and its actual semantic meaning. The calculation formula is: $\:FRIR=\frac{1}{N}\sum\:_{i=1}^{N}\mathbb{I}\left[{\boldsymbol{I}\boldsymbol{F}}_{i}\right]=\frac{{N}_{\mathrm{I}\mathrm{F}}}{N}$

**Failure Rate of Name Recognition (FRNR)**: This metric measures the proportion of cases where the API name output in the [Action] stage does not match the target API, under the premise that the model has correctly identified the calling intent. This metric only counts cases where $\:{f}_{i}\in\:\mathcal{F}$ but $\:{f}_{i}^{\ast\:}\ne\:{f}_{i}^{\ast\:}$ (i.e., the API exists but is not applicable to the current task). The calculation formula is:$\:FRNR=\frac{1}{N}\sum\:_{i=1}^{N}\mathbb{I}[{f}_{i}\ne\:{f}_{i}^{\ast\:}\wedge\:{f}_{i}\in\:\mathcal{F}]$

**Failure Rate of Parameter Configuration (FRPR)**: This metric measures the proportion of cases where the parameters constructed in the [Action Input] stage do not comply with API specifications (denoted as $\:{PF}_{i}$), under the premise that the model has correctly selected the API ($\:{f}_{i}={f}_{i}^{\ast\:}$). Parameter failures include: missing mandatory parameters, type errors, out-of-range values, and non-compliance with the JSON Schema format. The calculation formula is: $\:FRPR=\frac{1}{N}\sum\:_{i=1}^{N}\mathbb{I}[{f}_{i}={f}_{i}^{\ast\:}\wedge\:P{F}_{i}]$

##### Failure rate of API hallucination (FRHR)

This metric measures the proportion of hallucinatory outputs generated by the model, which is strictly defined as the output function name is not exists in the system-registered tool set $\mathcal{F}$.

The calculation formula is: $\:FRHR=\frac{1}{N}\sum\:_{i=1}^{N}\mathbb{I}[{f}_{i}\notin\:\mathcal{F}$

#### Logical relationships and mutual exclusivity among metrics

The above five metrics follow a hierarchical judgment logic to ensure that each sample is counted in only one failure category. For any sample, the judgment process is as follows: First, check for hallucinatory outputs (FRHR); if no hallucination exists, check the correctness of intent recognition (FRIR); if the intent is correctly recognized, check the matching of the API name (FRNR); if the name is correct, check the validity of parameter configuration (FRPR); only when all checks are passed is the sample included in the numerator of ACAR. This hierarchical structure ensures the mutual exclusivity of the metrics: a sample is never counted in both FRNR and FRHR, because FRNR only counts cases where a real but incorrect API is called, while FRHR counts cases of fictional APIs, refused calls, or empty calls.

#### Verification method based on the ReAct reasoning chain

To track the API calling behavior of LLMs, we construct an end-to-end visual verification system based on the ReAct (Reasoning and Acting) framework. The ReAct framework structures the model’s reasoning process into a cyclic chain of [Thought1]→[Action]→[Action Input]→[Observation]→[Thought2], and the output of each link is recorded for metric calculation.

Specifically, the output of the [Thought1] stage is used to determine FRIR: intent recognition is marked as failed if the model’s reasoning content does not involve any API calling intent, or if there is a fundamental deviation between the model’s understanding of the question and the expected semantic meaning. The output of the [Action] stage is used to determine FRNR and FRHR: we check whether the output function name exists in the system-registered tool set $\mathcal{F}$; if not, it is marked as a fictional API, and if it exists but does not match the target API, it is marked as a name recognition failure. The JSON parameters in the [Action Input] stage are used to determine FRPR: we validate the parameters using a predefined JSON Schema, checking the completeness of mandatory fields, consistency of data types, and validity of value ranges. The tool return results in the [Observation] stage are used to finally confirm whether the API is executed correctly: if valid data is returned, the call is included in the positive sample statistics of ACAR.

#### Judgment criteria and ilustrative examples

Table 4 presents the judgment criteria and specific examples for various failure types.

#### Implementation of the Verification Process

In practical verification, this study designed an end-to-end test module based on the LangChain framework, which inherits from the AgentExecutor of LangChain.

The test process is as follows: First, professional meteorological questions are input into the Agent system. Second, the large language model generates a reasoning process (Thought) to analyze the question requirements and formulate a solution strategy. Third, the model determines the action to be executed (Action), including the name of the selected tool function. Fourth, the model constructs function calling parameters (Action Input) in JSON format. Fifth, the Agent executor invokes the corresponding tool function and obtains the return result (Observation). Sixth, the model continues reasoning based on the observation result and decides whether to call more tools or generate a final answer. The entire process allows multiple iterative rounds until the model judges that sufficient information has been collected to answer the question.

After each round of model reasoning, the fields of [Thought1], [Action], [Action Input], [Observation] and [Thought2] are parsed automatically and matched against the predefined tool registration table F (consisting of 35 meteorological APIs). A logging system records the complete reasoning chain of each test sample in detail, including the original input, intermediate steps and final output, which supports post-hoc audit and error analysis. For 70 screened question-answer pairs, the above five metrics were calculated one by one, and the results were aggregated to obtain the overall performance evaluation of the model on the Function Calling task in the meteorological domain.[Thought2] Generate the correct answer based on the user’s intent and the API execution results.

## Experimental evaluation

### Baseline

We compare KG2data with RAG2data and chat2data. RAG2data replaces the knowledge graph module of the KG2data system with a vector database, which is constructed using the same data as KG2data. chat2data, on the other hand, removes the knowledge graph module from the KG2data system and only includes certain functional descriptions of the API within the prompt.

### Accuracy on API calls

In this study, a total of 70 meteorological instructions were employed to conduct API call experiments. Considering the limited sample size and the proportional nature of the experimental metrics, the Mann-Whitney U test (a non-parametric test for two independent samples) was adopted to verify the significance of performance differences between methods. Table 1 reports the performance of our method and baseline methods in meteorological API calls, with significance markers directly derived from the above statistical test results. Evidently, our method outperforms all baseline methods, achieving a higher ACAR and lower FRIR, FRNR, FRPR, and FRHR, with all performance improvements supported by statistical significance tests Tables 2, 3 and 4.

**Table 1:** Performance comparison of KG2data, RAG2data, and chat2dataNotes: ** denotes a statistical significance level of *p* < 0.05, and * denotes *p* < 0.1. The null hypothesis of the Mann-Whitney U test is that “there is no statistically significant difference in the performance of the two compared methods on the corresponding metric”. Row 4 presents the significance results of KG2data versus RAG2data, and Row 6 presents those of KG2data versus chat2data. A “trial” in the statistical test is defined as one API call task executed for a single meteorological instruction.

KG2data	FRIR	FRNR	FRPR	FRHR	ACAR
	0.00%	1.43%	2.86%	0.00%	88.57%
RAG2data	8.57%	16%	10.00%	10.00%	72.14%
	**	**	**	**	**
chat2data	1.43%	7.14%	7.14%	8.57%	71.43%
		*		**	**

**Table 2:** Meteorological-Related Entity Information.

Entity	Example Entities
Atmospheric System	Subtropical high, typhoon, shear line
Weather Phenomenon	Rainstorm, hailstorm, heavy fog
Physical Quantity	Water vapor flux, vorticity, air pressure
Location	Shandong Peninsula, Northwestern Shandong
Time	31 July 2005, flood season
Equipment	Doppler radar, wind profiler radar
Indicator	Showalter Index, K-Index
Formula	CAPE calculation formula
Event	2005 Shandong rainstorm event
Isobaric Surface	500 hPa, 850 hPa, 700 hPa

**Table 3:** Top 10 Most Frequent Relation Types.

Relation	Frequency	Example Triple
contains	108	(Vortex system, contains, cyclonic circulation)
belongs_to	58	(Rainstorm, belongs_to, severe weather)
has	54	(Typhoon, has, eye structure)
provides	44	(Radar, provides, reflectivity data)
used_for	43	(K-Index, used_for, severe convection forecasting)
influences	42	(Subtropical high, influences, precipitation distribution)
occurs_at	36	(Rainstorm, occurs_at, flood season)
indicates	26	(High CAPE value, indicates, severe convection potential)
uses	25	(Rainstorm diagnosis, uses, water vapor flux divergence)
appears_in	22	(Shear line, appears_in, 850 hPa)

Community Detection and Pruning.

**Table 4:** the judgment criteria and specific examples for various failure types.

Metric	Failure Type	Judgment Criteria	Example
FRIR	Intent Recognition Failure	[Thought1] fails to identify the need for an API call	User asks “Query the temperature in Beijing”; the model directly answers “It is sunny in Beijing today” without attempting to call any tool.
FRNR	Name Recognition Failure	[Action] calls a real but inapplicable API	User asks “Query the temperature in Beijing”; the model calls get_typhoon_info instead of get_2m_temperature.
FRPR	Parameter Configuration Failure	[Action Input] parameters do not comply with the Schema	When calling get_2m_temperature, the input is {“location”: “Beijing”} instead of {“city”: “Beijing”}.
FRHR-H1	Fictional API	[Action] outputs a non-existent function name	The model outputs get_water_vapor_flux_divergence, which is not available in the system.

**Fig. 4:**
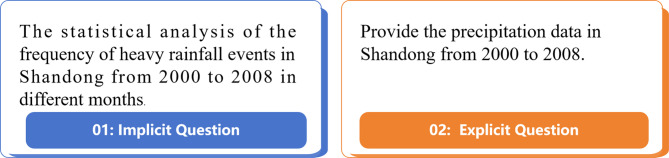
Implicit and explicit queries in the meteorological domain.

All metrics of RAG2data are inferior to those of KG2data. Compared with RAG2data, KG2data significantly improves the ACAR by 16.43% (Mann-Whitney U test, *p* < 0.05), and significantly reduces the FRIR, FRNR, FRPR and FRHR by 8.57%, 14.57%, 7.14% and 10.00%, respectively (all Mann-Whitney U test, *p* < 0.05). The knowledge graph endows KG2data with domain-specific meteorological reasoning capabilities, enabling it to effectively identify key information required to answer user queries—even when the queries are implicit, lengthy, or do not directly contain API-related details (see Example 1 in Fig. 4). Furthermore, the triple-based knowledge representation efficiently filters out irrelevant and redundant information, retaining only the core entities and relationships essential to the query. The accurate and professional domain knowledge provided by the knowledge graph not only significantly improves the FRIR and ACAR of LLMs in API call tasks (Mann-Whitney U test, *p* < 0.05), but also largely mitigates execution errors caused by LLM hallucinations, as evidenced by the significantly lower FRHR of KG2data compared to RAG2data (Mann-Whitney U test, *p* < 0.05). Although Retrieval-Augmented Generation (RAG) also provides background knowledge for RAG2data, such knowledge is retrieved from the vector database based on vector similarity. In the case of implicit queries, RAG may fail to provide useful information; moreover, for verbose queries, RAG typically retrieves a large volume of irrelevant information, which may trigger LLM hallucinations and impede the system’s ability to execute API calls effectively.

KG2data also exhibits significantly superior performance to chat2data: it significantly reduces the FRNR and FRHR by 5.71% (Mann-Whitney U test, *p* < 0.1) and 8.57% (Mann-Whitney U test, *p* < 0.05), respectively, and significantly improves the ACAR by 17.24% (Mann-Whitney U test, *p* < 0.05). Chat2data lacks domain-specific knowledge and demonstrates insufficient reasoning capabilities, which easily induces hallucinations in LLMs or makes it difficult to correctly identify the appropriate API, thus hindering the effective execution of API calls.

Notably, although the KG2data system can accurately determine which API to call during the [observation] step of the agent’s reasoning chain, LLM hallucinations lead to the absence of API call return parameters in the final answer. This finding indicates that the performance of LLMs and the design of prompt templates have a significant impact on the final answer. In future work, we plan to design more rigorous prompt templates to enhance the system’s performance in API call tasks. We also anticipate the release of more advanced LLMs with improved capabilities and hallucination mitigation.

## Discussion

### Analysis and discussion of the KG2data framework

To clarify the fundamental architectural differences between the proposed KG2data and the baseline systems (RAG2data and Chat2data), we first elaborate on their core structural designs and functional modules. Chat2data adopts a lightweight framework with only the core LLM and API functional description prompts, lacking any domain-specific knowledge enhancement module; it relies solely on the LLM’s inherent semantic understanding ability to map natural language queries to API calls, with no external knowledge retrieval or reasoning support. RAG2data optimizes Chat2data by introducing a Retrieval-Augmented Generation module, which constructs a vector database with domain data for knowledge retrieval—its architecture consists of a knowledge management layer (for data preprocessing and vector storage), an online query inference layer (for vector-based retrieval and intent analysis), and an LLM layer (for result generation). However, RAG2data only realizes shallow knowledge matching through vector similarity search, without structured knowledge representation and domain reasoning capabilities. In contrast, KG2data in tihs study reconstructs the knowledge enhancement layer with a domain-specific meteorological knowledge graph (instead of a vector database) and integrates a ReAct-expert Agent as the core task scheduling module; its four core components (meteorological knowledge graph, ReAct-expert Agent, LLM, and data acquisition tools) form a closed loop of “knowledge reasoning-task decomposition-API calling-result verification”, realizing deep integration of structured domain knowledge and step-by-step reasoning for API call tasks.

Besides, the KG2data devoted to address the challenges associated with the underutilization of meteorological datasets and the limitations of LLM-driven API calls in specialized domains due to knowledge gaps. The KG2data framework demonstrates superior performance in accuracy of API call. We compare the proposed framework with RAG2data and chat2data. The results indicate that KG2data is more robust to prompt perturbations and achieves significantly higher in ACAR and lower in FRNR for API calls. Moreover, it reduces FRHR in API calls caused by LLM’ hallucinations. We hypothesize that the improvement in performance is due to the fusion of explicit knowledge from the knowledge graph, sophisticated architectures from React-expert agents, and intent-recognition capabilities of the LLM. These findings highlight the value of providing fine-grained domain-specific knowledge at the prompt and emphasize the significance of designing reasoning-behavior-interleaved expert agents for guiding LLM in executing precise tasks.

It is important to note that knowledge graphs significantly improve the performance of LLM in domain-specific API call tasks. In this study, the integration of heterogeneous, domain-specific knowledge into the knowledge graph holds the potential to generate new insights by linking different entities^43^. Knowledge-augmented LLM are able to produce reliable, comprehensive responses rooted in domain-specific expertise. Additionally, the reasoning capabilities of knowledge graphs in specialized fields enhance both the accuracy of LLM responses and their performance in handling complex queries. This forms the basis for enabling agents to accurately identify the most relevant APIs for user queries^44^. The structured framework of knowledge graphs also greatly strengthens the effectiveness of prompts^45^, as the structured format allows for exponential growth in entity connections, making the injected knowledge far more information-dense than RAG.

In terms of architectural design, the KG2data system employs a modular, loosely coupled, and highly cohesive approach, dividing the system into distinct modules, each responsible for specific functions and tasks. This design philosophy enhances both scalability and maintainability.The KG2data system is built on a systems engineering framework, with a set of unified standards and specifications. It integrates a range of technologies, including data tools developed through domain-specific platform APIs, intelligent agent workflows, large model generation, and knowledge graphs. These integrations provide the system with high availability, smooth scalability, and rapid deployment capabilities.

In conclusion, our framework highlights the significance of combining LLM, knowledge graph, and ReAct-master expert agents, which greatly reduces the difficulty and cost for non-experts in accessing specialized data. However, due to time and resource limitations, the KG2data system has only been tested with 35 virtual APIs and 70 instruction-answer pair. Moving forward, we aim to explore a broader range of domain-specific API calls.

### Limitations of the KG2data framework

Although the KG2data method outperforms the baseline methods in benchmark tests, the following key limitations should be acknowledged when interpreting the conclusions.This study adopts simulated virtual meteorological APIs, which do not cover engineering characteristics in real-world scenarios such as network latency and authentication failures. The framework needs to be optimized to adapt to production-grade API environments. The synthetic question-answer pairs used for evaluation are constructed by experts with standardized expressions and low noise, which cannot restore the colloquial and ambiguous features of real meteorological queries and may overestimate the actual robustness of the model. Meanwhile, the small-scale benchmark set with only 70 test cases and 35 tool functions limits the statistical power of the conclusions. Furthermore, the experiments focus solely on the single meteorological domain, and no cross-domain generalization inferences are made temporarily.In summary, KG2data achieves superior performance in synthetic tests, but its practical deployment and cross-domain application value still require further verification on real meteorological APIs, real user queries and large-scale task sets.

## Conclusion

This study introduces the KG2data system, which enables the intelligent and automated retrieval of meteorological data through a conversational interface. The system overcomes the limitations of traditional API calls, which often lack domain-specific knowledge and tend to have low accuracy in specialized scenarios, such as data analysis. It also addresses the challenge of interpreting professional users’ questions, which can be implicit or overly complex, making successful API calls difficult. The framework LLM, knowledge graph, ReAct-expert agent, and tool-utilization technologies. In this framework, the LLM (for reasoning and semantic understanding) complement the knowledge graph (which provides domain-specific knowledge, reasoning, and complex query capabilities), enhancing their combined effectiveness. The ReAct-master agent facilitates task decomposition and precise execution through an interwoven reasoning-action process. Specifically, the [Action] step identifies the API most relevant to the user’s query, the [Action Input] step provides the necessary input parameters, and the [Observation] step presents the results of the API calls. This step-by-step breakdown effectively clarifies the reasoning process behind API calls in LLM, enabling easier identification of potential issues that could cause API calls failures.

Furthermore, this system addresses several limitations of RAG when applied to large-scale datasets, such as its inability to extract deep semantic associations, incomplete coverage in retrieval, difficulty in integrating data from multiple sources, delayed data updates, limited adaptability to complex query tasks, and the lack of reasoning and expansion for specialized domain knowledge. In contrast, knowledge graphs store knowledge in a graph structure, which clearly represents the complex relationships between entities, enabling LLM to comprehensively integrate and understand domain-specific knowledge. RAG, on the other hand, primarily relies on vector similarity for retrieval, making it less effective in uncovering deep semantic relationships. Knowledge graphs can integrate data from diverse sources, including structured, semi-structured, and unstructured data, into a unified knowledge graph. In contrast, RAG requires adaptation and integration of data from multiple sources, limiting its capability for complex queries. Knowledge graphs support complex queries based on graph structures, such as path queries and subgraph queries, facilitating various complex tasks in specialized domains. Conversely, RAG primarily supports keyword-based queries, limiting its ability to handle complex queries effectively. Additionally, knowledge graphs enable reasoning and expansion for domain-specific knowledge, while RAG lacks reasoning capabilities and mainly relies on the inherent reasoning abilities of LLM.

In conclusion, the KG2data framework we propose will significantly enhance the performance of LLM in specialized domain API applications. It also effectively addresses the challenges of high cost of training and fine-tuning for LLM, as well as their struggle to adapt to the dynamic changes in APIs and domain-specific knowledge. The KG2data framework shows great potential for improving the efficiency of data processing and utilization in specialized fields.

## Data Availability

The datasets used and/or analysed during the current study are available from the corresponding author on reasonable request.

## References

[1] AghaKouchak, A. et al. Climate Extremes and Compound Hazards in a Warming World. In: Jeanloz R, Freeman KH, editors. Annual Review of Earth and Planetary Sciences, Vol 48, 2020. Annual Review of Earth and Planetary Sciences, 519 – 48. (2020).

[2] Ebi, K. L. et al. Extreme Weather and Climate Change: Population Health and Health System Implications. In: Fielding JE, editor. Annual Review of Public Health, Vol 42, 2021. Annual Review of Public Health, 293–315. (2021). 10.1146/annurev-publhealth-012420-105026 PMC9013542 33406378

[3] Sindall, R. et al. Drowning risk and climate change: A state-of-the-art review. *Inj. Prev.* **28**(2), 185–91. 10.1136/injuryprev-2021-044486 (2022). 35197275 10.1136/injuryprev-2021-044486 PMC8938664

[4] Cabaneros, S. M., Calautit, J. K. & Hughes, B. R. A review of artificial neural network models for ambient air pollution prediction. *Environ. Model. Softw.* **119**, 285–304. 10.1016/j.envsoft.2019.06.014 (2019).

[5] Slater, L. J. et al. Hybrid forecasting: Blending climate predictions with AI models. *Hydrol. Earth Syst. Sci.* **27**(9), 1865–89. 10.5194/hess-27-1865-2023 (2023).

[6] Yadav, A. K. & Chandel, S. S. Solar radiation prediction using Artificial Neural Network techniques: A review. *Renew. Sustain. Energy Rev.* **33**, 772–81. 10.1016/j.rser.2013.08.055 (2014).

[7] Sun, Y. et al. Deep learning in statistical downscaling for deriving high spatial resolution gridded meteorological data: A systematic review. *Isprs J. Photogramm. Remote Sens.* **208**, 14–38. 10.1016/j.isprsjprs.2023.12.011 (2024).

[8] Qin, Y., Wang, Y., Zhang, B. & Fan, W. Research and implementation of Provincial Meteorological BigData Service Platform Based on Extranet. *Meteorol. Sci. Technol.* **48**(06), 823–54. 10.19517/j.1671-6345.20190546 (2020).

[9] Frazier, A. E. & Hemingway, B. L. A technical review of Planet Smallsat data: Practical considerations for processing and using PlanetScope imagery. *Remote Sens.*10.3390/rs13193930 (2021).

[10] Belward, A. S. & Skoien, J. O. Who launched what, when and why; Trends in global land-cover observation capacity from civilian earth observation satellites. *Isprs J. Photogramm. Remote Sens.* **103**, 115–128. 10.1016/j.isprsjprs.2014.03.009 (2015).

[11] Yu, X., Chai, C., Li, G. & Liu, J. Cost-based or learning-based? A hybrid query optimizer for query plan selection. *Proc. VLDB Endow.* **15**(13), 3924–3936. 10.14778/3565838.3565846 (2022).

[12] Sun, Z., Zhou, X. & Li, G. Learned index: A comprehensive experimental evaluation. *Proc. VLDB Endow.* **16**(8), 1992–2004. 10.14778/3594512.3594528 (2023).

[13] Zhou, X., Li, G., Feng, J., Liu, L. & Guo, W. Grep: A graph learning based database partitioning system. *Proc ACM Manag Data*10.1145/3588948 (2023).

[14] Tang, N. et al. RPT: Relational pre-trained transformer is almost all you need towards democratizing data preparation. *Proc. VLDB Endow.* **14**(8), 1254–61. 10.14778/3457390.3457391 (2021).

[15] Zhao, F. et al. LLM-SQL-Solver: Can LLMs Determine SQL Equivalence? 2025 IEEE International Conference on Big Data (BigData), 1887–1894. 10.1109/BIGDATA66926.2025.11401595 (2025).

[16] Zhou, X., Sun, Z. & Li, G. DB-GPT: Large language model meets database. *Data Science and Engineering* **9**(1), 102–11. 10.1007/s41019-023-00235-6 (2024).

[17] Sallam, M. ChatGPT utility in healthcare education, research, and practice: Systematic review on the promising perspectives and valid concerns. *Healthcare*10.3390/healthcare11060887 (2023). 36981544 10.3390/healthcare11060887 PMC10048148

[18] Samsi, S. et al. From Words to Watts: Benchmarking the Energy Costs of Large Language Model Inference. IEEE High Performance Extreme Computing Virtual Conference (HPEC). Electr Network. (2023).

[19] Schick, T. et al. Toolformer: language models can teach themselves to use tools. Proceedings of the 37th International Conference on Neural Information Processing Systems. New Orleans, LA, USA: Curran Associates Inc. p. Article 2997. (2024).

[20] Thoppilan, R. et al. LaMDA: Language Models for Dialog Applications. ArXiv abs/2201.08239. (2022).

[21] OpenAi, Achiam, J. et al. GPT-4 Technical Report. (2023). 10.48550/arXiv.2303.08774

[22] Liang, Y. et al. TaskMatrix.AI: Completing tasks by connecting foundation models with millions of APIs. *Intelligent Computing* **3**, 0063. 10.34133/icomputing.0063 (2024).

[23] Zhao, X., Zhou, X. & Li, G. Chat2Data: An interactive data analysis system with RAG, vector databases and LLMs. *Proc. VLDB Endow.* **17**(12), 4481–4484. 10.14778/3685800.3685905 (2024).

[24] Soman, K. et al. Biomedical knowledge graph-optimized prompt generation for large language models. *Bioinformatics*10.1093/bioinformatics/btae560 (2024). 39288310 10.1093/bioinformatics/btae560 PMC11441322

[25] Oladeji, O., Mousavi, S. S. & Roston, M. AI-driven E-liability knowledge graphs: A comprehensive framework for supply chain carbon accounting and emissions liability management. *Arxiv*https://doi.org/arXiv:2312.00045 (2023).

[26] Arsenyan, V., Bughdaryan, S., Shaya, F., Small, K. & Shahnazaryan, D. Large language models for biomedical knowledge graph construction: Information extraction from EMR notes. *Arxiv* https://doi.org/arXiv:2301.12473 (2023).

[27] Sun, Q. et al. Docs2KG: Unified Knowledge Graph Construction from Heterogeneous Documents Assisted by Large Language Models. arXiv preprint arXiv:2406.02962 (2024).

[28] Lairgi, Y. et al. iText2KG: Incremental knowledge graphs construction using large language models. *Lect. Notes Comput. Sci.* 15439, 214–229. 10.1007/978-981-96-0573-6_16 (2025).

[29] Wang, J. et al. Learning to Plan for Retrieval-Augmented Large Language Models from Knowledge Graphs. *Findings Assoc. Comput. Linguist.: EMNLP 2024,* 8271–8283. 10.18653/v1/2024.findings-emnlp.459 (2024).

[30] Lan, Y. et al. Path-based knowledge reasoning with textual semantic information for medical knowledge graph completion. *BMC Med. Inform. Decis. Mak.*10.1186/s12911-021-01622-7 (2021). 10.1186/s12911-021-01622-7 PMC8628388 34844576

[31] Zhu, Y. et al. DualDE: Dually Distilling Knowledge Graph Embedding for Faster and Cheaper Reasoning. 15th ACM International Conference on Web Search and Data Mining (WSDM). Electr Network1516-24. (2022).

[32] Schneider, P. et al. Evaluating large language models in semantic parsing for conversational question answering over knowledge graphs. *Lect. Notes Comput. Sci.* **15592**, 117–133. 10.1007/978-3-031-87329-4_8 (2025).

[33] Huang, J., Parthasarathi, P., Rezagholizadeh, M. & Chandar, S. Towards Practical Tool Usage for Continually Learning LLMs. arXiv preprint arXiv:240409339. (2024).

[34] Zhang, K., Chen, H., Li, L. & Wang, W. Syntax error-free and generalizable tool use for llms via finite-state decoding. arXiv preprint arXiv:231007075. (2023).

[35] Tufano, R. et al. Unveiling ChatGPT’s Usage in Open Source Projects: A Mining-based Study. 2024 IEEE/ACM 21st International Conference on Mining Software Repositories (MSR): IEEE. pp. 571 – 83. (2024).

[36] Mo, F. et al. A Survey of Conversational Search. *ACM Trans. Inform. Syst.* **43** (6), 1–50 (2025).

[37] Inan, E. Contrastive retrieval methodology for Turkish metaphor detection and identification. *ACM Trans. Asian Low-Resour. Lang. Inf. Process.*10.1145/3770072 (2025).

[38] Patil, S. G., Zhang, T., Wang, X. & Gonzalez, J. E. Gorilla: Large language model connected with massive apis. arXiv preprint arXiv:230515334. (2023).

[39] Shen, Y. et al. HuggingGPT: Solving AI tasks with ChatGPT and its friends in Hugging Face. *Adv. Neural. Inf. Process. Syst.* **36**, 10.48550/arXiv.2303.17580 (2023).

[40] Escarda-Fernández, M. et al. *LLMs on the Fly* (Text-to-JSON for Custom API Calling, 2024).

[41] Healy, K., Srinivasan, B., Madathil, V. & Wu, J. Internal Representations as Indicators of Hallucinations in Agent Tool Selection. arXiv preprint arXiv:2601.05214v1 [cs.AI], 1–31. 10.48550/arXiv.2601.05214 (2026).

[42] Wang, Y. et al. Self-instruct: Aligning language models with self-generated instructions. arXiv preprint arXiv:221210560. (2022).

[43] Baranzini, S. E. et al. A biomedical open knowledge network harnesses the power of AI to understand deep human biology. *AI Mag.* **43**(1), 46–58. 10.1002/aaai.12037 (2022). 36093122 10.1002/aaai.12037 PMC9456356

[44] Yasunaga, M. et al. QA-GNN: Reasoning with Language Models and Knowledge Graphs for Question Answering. Conference of the North-American-Chapter of the Association-for-Computational-Linguistics - Human Language Technologies (NAACL-HLT). Electr Network535-46. (2021).

[45] Pan, J. Z. et al. Large Language Models and Knowledge Graphs: Opportunities and Challenges. Arxiv. doi: arXiv:2308.06374. (2023).

